# Mr. Pulley's Case of Amputation at the Shoulder-Joint

**Published:** 1805-08-01

**Authors:** John Pulley

**Affiliations:** Bedford


### *To the Editors of the Medical and Physical Journal.*

Gentlemen,

As amputation at the shoulder-joint has been performed but seldom, perhaps the relation of the following case may be acceptable to your readers.

Sturgeon Church, about forty years of age, of thin and scrophulous habit, was admitted into the Bedford Infirmary in October last. About six months previous to her admission, she was attacked with pain under the deltoid muscle, which resisted the usual applications for relief; and a few weeks subsequently, an abscess was discovered, pointing about four inches below the shoulder-joint, on the posterior part of the arm; this abscess was punctured, and continued to discharge on her admission into the infirmary. At the period of her admission, she complained of pain shooting from the acromion, downward to about the insertion of the deltoid muscle; her arm was perfectly useless; the motion of the joint was very much impeded, and on every movement the existence of carious bone was discoverable. The superincumbent parts of the joint were much thickened; and from the touch and motion of the limb, I was led to believe that the disease existed chiefly about the head of the humerus. On passing a probe within the orifice of the abscess, I could direct it upward to near the acromion, but never apparently within the capsular ligament. The patient remained for two months under the trial of external and internal remedies without receiving any benefit; the pain and discharge continued unabated, and symptoms of irritation were daily and seriously increasing; from this state, it was evident that death would be the consequence, and that the only chance of preserving life, rested on the removal of the limb; it was agreed in consultation that this step should be prosecuted, and to which the patient consented. I must beg leave to state in what way the operation was performed. The patient being placed in a proper recumbent position, and the subclavian artery being securely compressed in its passage over the first rib, I began my first incision between the head of the humerus and the chest, nearly in a line with the glenoid cavity of the scapula, and carried it downward through the integuments to the under part of the arm, about opposite to the insertion of the deltoid muscle. Having done this, I divided the pectoral muscle upward, when I dissected sected down to the brachial artery, laying it bare to the extent of more than an inch, and having the arteria profunda in view; I then passed a needle and ligature under the vessel, and secured it above the profunda. The danger of hæmorrhage being now removed, I made my circular incision directly down to the bone, corresponding with the inferior point of the longitudinal incision; I then made a second longitudinal incision at once to the bone, through the centre of the deltoid muscle, from the acromion down to the circular cut, when I dissected back the flaps, and removed the limb by cutting around the capsular ligament, and dividing the tendon of the long head of the biceps muscle. The large muscular branches of arteries were taken up as divided, and not more than eight ounces of blood were lost during the operation.

Mr. Benjamin Bell, in his instructions on this operation, has advised the circular incision to be first made, and the brachial artery to be taken up by the aid of the tenaculum; he maintains, that by this method the operation is more simplified, rendered less painful, and the danger of hæmorrhage diminished; also, that more muscular branches of arteries are preserved for the nourishment of the stump.

Notwithstanding such high authority, it appears to me that security is best promoted by tying the artery at the commencement of the operation; by this mode, the ligature is less likely to be loosened or thrown off by the muscular and elastic action of the vessel; and the artery is less likely to be wounded in dissecting up the inner flap; as to the preservation of more muscular branches, they must ever be sufficiently numerous to afford nourishment for the stump, and to promote the healing process.

The head of the bone was not enlarged, as I had reason to expect; but its whole articulatory surface was in a rough, diseased state; the glenoid cavity of the scapula appeared much less injured, its roughness being chiefly confined along its inner edge; the point of the acromion had a slight degree of roughness.

The poor woman bore the operation with unexampled fortitude, almost without a single expression of pain; and after it, she remained in a very tranquil state.

On the sixth day the dressings were removed, and the stump put on a very favourable appearance; the integuments and muscles seemed mostly to have healed by the first intention; the pain was less, and the discharge better in quality, and not greater than previous to the operation.

On the eighth day, she complained of much uneasiness above above the supra spinatus muscle; and on examination, there appeared a large collection of pus beneath the integuments.

On the following day, the abscess was punctured, and more than two ounces of matter discharged; the probe was used, but no roughness of the scapula could be discovered. This abscess continued daily to suppurate freely, but still the hectic symptoms were abated, and her strength seemed rather to increase.

At the expiration of a fortnight, I regret to say that my patient altered materially for the worse; her hectic symptoms returned, accompanied with pain about the chest, cough, and an apparent expectoration of pus; she grew gradually weaker, and died on the twenty-fourth day after the operation, from sudden hæmorrhage.

On examining the parts, I found that the healing process had advanced more rapidly than could have been expected; no sloughy appearance existed, but the spine of the scapula in several parts was in a rough state. The ligatures of the muscular branches of arteries were all removed within eight days subsequent to the operation; a fortnight elapsed before the principal ligature was touched; it was then every day slightly lifted with the greatest caution, until the day previous to her decease; this ligature was still found retained, the artery being nearly ulcerated through, without its diameter being in the least contracted. Within the orifice of the artery, a coagulum was loosely attached, about half an inch in length, and not larger in circumference than the body of a barley corn. On opening the thorax, the lungs were found in a very diseased state; many tubercles existed, and ulcerations were extensive. About four ounces of water were found in the pericardium. On the right side of the thorax, between the pleura pulmonalis and pleura costalis, a sac existed, containing not less than a pint and a half of watery fluid; this sac possessed a white, shining appearance, and was much firmer than the usual envelope of hydatids. Could this diseased state of the thorax have been previously ascertained, it would have been highly unjustifiable to have proposed an operation so severe in execution, and so precarious in issue; but, singular as it may seem, no symptoms existed, indicating a collection of water, or an ulcerated state of the lungs; the pulse of the patient had no irregularity, and she had no difficulty in lying on either side; and previous to the operation, she had no expectoration. Here it is right to remark, that a few few days after her admission into the Infirmary, she was attacked with pain in the chest, accompanied by cough; and which was succeeded by a frothy, mucous expectoration; but for some time previous to the operation, her pain, cough, and expectoration were altogether removed. If this formidable diseased condition of the thorax had not existed, and a coagulum had formed sufficiently firm, on the ulceration of the artery, to have resisted the impetus of the blood, there is some reason to believe that this woman would have ultimately recovered the carious state of the scapula.

A state of rest is the principal consideration to enable the blood to coagulate within its vessels; and this state it is very difficult to obtain in the larger arteries, where the quantity of the circulating fluid is great, and muscular and elastic power are vigorous in action.

After amputation at the shoulder-joint, would it not be advisable, to assist the coagulation of the blood, to have firm pressure made on the subclavian artery in its passage over the first rib, for an hour or more, twice or thrice daily, till the ligature be disengaged by the ulceration of the vessel? I am, &c.

*Bedford, May* 12, 1805. JOHN PULLEY.

